# Case report: A late-onset cobalamin C defect first presenting as a depression in a teenager

**DOI:** 10.3389/fgene.2022.1012558

**Published:** 2022-10-20

**Authors:** Siqi Cheng, Weihong Chen, Mingmin Zhao, Xing Xing, Lei Zhao, Bowen Ren, Na Li

**Affiliations:** ^1^ Department of Neurology, Hebei General Hospital, Shijiazhuang, China; ^2^ Graduate School, Hebei North University, Chengde, China

**Keywords:** CblC defect, MMACHC, depression, genotype–phenotype correlation, case report

## Abstract

**Background:** The cobalamin C (cblC) defect, a common inborn disorder of cobalamin metabolism due to a genetic mutation in *MMACHC*, can cause combined methylmalonic acid and homocysteine accumulation in blood, urine, or both. In this article, a late-onset case was reported, and the patient first presented with depression identified with the *MMACHC* gene. We summarized the clinical features of the cblC defect, the relationship between genotype and phenotype, and the clinical experience concerning the diagnosis and treatment of the cblC defect.

**Case presentation:** Initially presented with depression, the 16-year-old female patient showed progressive abnormal gait and bilateral lower limb weakness after 3 months. Blood routine examination suggested severe hyperhomocysteinemia, and screening for urine organic acids found elevated methylmalonic acid. Family gene sequencing showed mutations detected in *MMACHC*. She had a compound heterozygous mutation, while the c.271dupA (p.R91Kfs∗14) was only detected in her father and the c.482 G>A (p.R161Q) was only detected in her mother. Hence, she was diagnosed with a cblC defect and treated with B vitamin supplements. The muscle strength of both lower limbs improved notably.

**Conclusion:** This case indicated that depression could be a presenting sign of cblC-type methylmalonic aciduria and homocysteinemia, and enhanced the genotype–phenotype relationship of the cblC defect, which will contribute to further understanding of this emerging disease.

## Background

Cobalamin C defect (cblC, OMIM 277400), a kind of complementation group disease of defective cblC metabolism described by Rosenberg in 1975 (Gravel et al., 1975), can result in both methylmalonic acid (MMA) and homocysteine (Hcy) accumulation *in vivo*, which was first reported by professor Mudd in 1969 (Mudd et al., 1969). It is an inherited metabolic disease caused by a mutation in *MMACHC* (OMIM 609831) (Lerner-Ellis et al., 2006). A CblC defect is the most common genetically inherited reason for combined methylmalonic aciduria and homocysteinemia, constituting the main biochemical phenotype of China and accounting for 60%∼80% of the domestic methylmalonic aciduria (Liu et al., 2012; Zhang et al., 2007).

The clinical features of the cblC defect vary by age of disease onset. Differing from the early-onset type (<1 year old), the late-onset cases (>4 years old) usually have milder clinical manifestations and a better prognosis after early intervention (Martinelli et al., 2011).

Sometimes there is a failure to detect cblC defects early due to their heterogeneous phenotypic spectrum of presentations. Normally, the cblC defect produces a series of multisystem neurological symptoms, such as psychological and behavioral abnormalities, leukoencephalopathy, encephalopathy, subacute combined degeneration of the spinal cord, lower extremity weakness, gait disorder, and seizures (Martinelli et al., 2011; Carrillo-Carrasco et al., 2012). However, there are few case reports focusing on cblC patients initially manifesting as depression. Here, we reported one novel late-onset cblC case, where the patient first presented with depression, carrying heterozygous variants in *MMACHC*, and also explored the pathophysiological mechanism. To date, only a small number of studies have found a potential association between the cblC genotype and its phenotype, which can help predict the age of disease onset and severity (Matos et al., 2013; Yu et al., 2015). Therefore, more comprehensive studies on the clinical and genetic features of cblC patients are warranted.

## Case description

The 16-year-old girl was born at full term with a spontaneous delivery from a non-consanguineous Chinese Han family. Her parents were healthy and denied a family history of neurologic illness. The girl first suffered from low mood, low self-esteem, self-blame, and visual hallucinations. Additionally, the neuropsychological scale at admission, particularly self-reporting inventory-90 and patient health questionnaire-9, pointed toward the occurrence of depression. A routine blood examination suggested moderate iron-deficiency anemia (hemoglobin of 86 g/L) and moderate homocysteinemia (Hcy of 62 μmol/L, reference range: 5–15 μmol/L). Brain MRI showed patchy and symmetric white matter hyperintensities in the posterior horn of the bilateral lateral ventricles. She was diagnosed with depression by her psychiatrist and was orally administered antidepressants, iron, and methylcobalamin (Mecbl). The depression and hallucination symptoms were alleviated after pharmacological therapy for only 2 weeks.

After 3 months, she showed gradually progressive abnormal gait and bilateral lower limb weakness without depression relapse, and she could hardly even stand without assistance. Physical examination also revealed hyperactive reflexes, bilateral ankle clonus, a positive Babinski sign, and suspicious deep sensory impairment. Iron-deficiency anemia and homocysteinemia were still detected with hyperuricemia. A review of the cerebral MRI found that the previous lesions displayed no change and were not enhanced on the spoiled gradient recalled (SPGR) sequence. The results of the special blood test were negative for pepsinogen I and II, pro-gastrin-releasing peptide, immune, and rheumatic factors. Moreover, neither serum nor cerebrospinal fluid discovered positive results of myelin oligodendrocyte glycoprotein antibody, aquaporin 4 antibody, glial fibrillary acidic protein antibody, and autoimmune encephalitis-related auto-antibodies.

**FIGURE 1:**
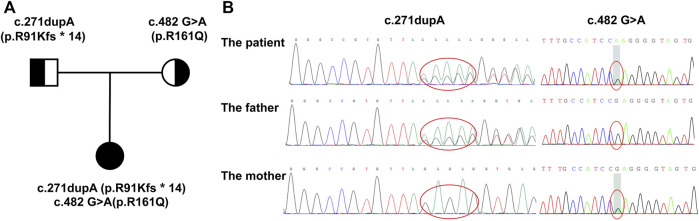
Family pedigree displaying the mutations detected in *MMACHC*. **(A)** The pedigree of the family with cblC defects. The proband is the daughter, and her parents have no signs of MMA. **(B)** Mutations detected in the family. The proband has a compound heterozygous mutation, while c.271dupA was inherited from her mother and c.482 G>A was from her father.

After excluding other diseases leading to multisystem involvement, inborn metabolic diseases were considered. The detailed information of this patient is shown in Table 1. Screening for urine organic acids was performed after 3 months of cobalamin supplement and still, elevated methylmalonic acid of 21.2 mmol/mol creatinine (reference range: 0.0–4.0 mmol/mol creatinine) was found. Amino acids and acylcarnitines analysis in blood were normal. Notably, with family whole exome sequencing and Sanger sequencing performed, it was detected that a compound heterozygous mutation existed in the *MMACHC* gene. One frameshift variant was c.271dupA (p.R91Kfs^∗^14), inherited from her mother, and the other variant, belonging to the missense type, was c.482 G>A (p.R161Q) from her father. According to the American College of Medical Genetics and Genomics (ACMG), both variants are pathogenic (Lerner-Ellis et al., 2006; Lerner-Ellis et al., 2009; Richards et al., 2015) (Figure 1).

**TABLE 1:** Clinical profiles of this patient.

	Test	Patient
Characteristic	Gender	Female
	Age of onset	16 years old
Clinical symptom	Growth	BMI = 15.76
	Psychiatric symptom	Depression and visual hallucinations
	Central nervous system	Bilateral lower limb weakness and abnormal gait
	Peripheral nervous system	Suspicious deep sensory impairment
Laboratory study	Red blood cell	Hemoglobin: 86 g/L
	Iron metabolism	Serum ferritin: 6.9 ng/ml, serum iron: 2.50 μmol/L, and unsaturated iron-binding capacity: 71.30 μmol/L
	Homocysteine	62 μmol/L
	Serum uric acid	452.7 μmol/L
	Pepsinogen	Pepsinogen I: 27.51 ng/ml, pepsinogen II: 13.42 ng/ml, pepsinogen I/II: 2.05, and pro-gastrin-releasing peptide: 44.63 pg/ml
	Rheumatic index	Antinuclear antibody, rheumatoid factor, rheumatic factor, and c-reactive protein (CRP) were negative
	Hematological index	Hematologic antibody cytokines, anti-human globulin antibodies (Coombs test), and paroxysmal nocturnal hemoglobinuria-related (PNH) antibodies were negative
	Autoimmune encephalitis	Negative
	Central nervous system demyelinating disease	Myelin oligodendrocyte glycoprotein (MOG) antibody and aquaporin 4 (AQP4) antibody were negative
Imaging	Electroencephalography	Normal
	Brain MRI	Patchy and symmetric white matter hyperintensities in the posterior horn of the bilateral lateral ventricles
Neurotic electrophysiology	No characteristic changes in the electroneurograms and somatosensory evoked potentials
CSF study	Pressure and routine test	Normal
	Biochemistry indicators	Normal
	Autoimmune encephalitis	Negative
	Central nervous system demyelinating disease	MOG antibody and AQP4 antibody were negative
	Autoimmune disease	Glial fibrillary acidic protein (GAFP) antibody was negative
	Microbiological and virological test	Normal
Screening for organic acids (after administration)	Urine organic acids	Methylmalonic acid of 21.2 mmol/mol creatinine (0.0–4.0 mmol/mol creatinine)
	Amino acids and acylcarnitine analysis in the blood	Normal
Gene sequencing	A compound heterozygous mutation in the *MMACHC* gene, c.271dupA (p.R91Kfs^∗^14), and c.482 G>A (p.R161Q)	
Diagnosis	Cobalamin C defect	

From the second onset, the patient got an intramuscular injection of vitamin B1 (50 mg/d) and cyanocobalamin (0.5 mg/d), intravenous injection of Mecbl (1 mg/d), and oral medication of vitamin B6 and folic acid tablets. The patient was not treated with betaine, for betaine is not available in our hospital. After 1 month of treatment, she could stand and walk for a short distance by herself; thus, she was allowed to be discharged from the hospital. The long-term oral administration of vitamin B and intermittent injections were executed after discharge. In the following 2 months, the muscles of both lower limbs gradually strengthened, so that she could walk indoors. The concentrations of MMA and Hcy were lowered.

## Discussion

As expected, the cblC defect is characterized by a heterogeneous clinical picture involving multipart nervous system symptoms, such as psychological and behavioral abnormalities, leukoencephalopathy, encephalopathy, and subacute combined degeneration of the spinal cord, extremity weakness, ataxia, and seizures. Notably, some studies have indicated that psychological and behavioral abnormalities are a presenting sign of late-onset cblC defect, among which schizophrenia and auditory hallucinations were common, rather than depression (Martinelli et al., 2011; Carrillo-Carrasco et al., 2012; Wei et al., 2019).

Our case report demonstrates that depression is also one of the initial manifestations of the late-onset cblC, which usually misleads psychologists or psychiatrists to diagnose it as a plain mood disorder. However, the mechanism of depression in cblC patients remains unclear.

**FIGURE 2:**
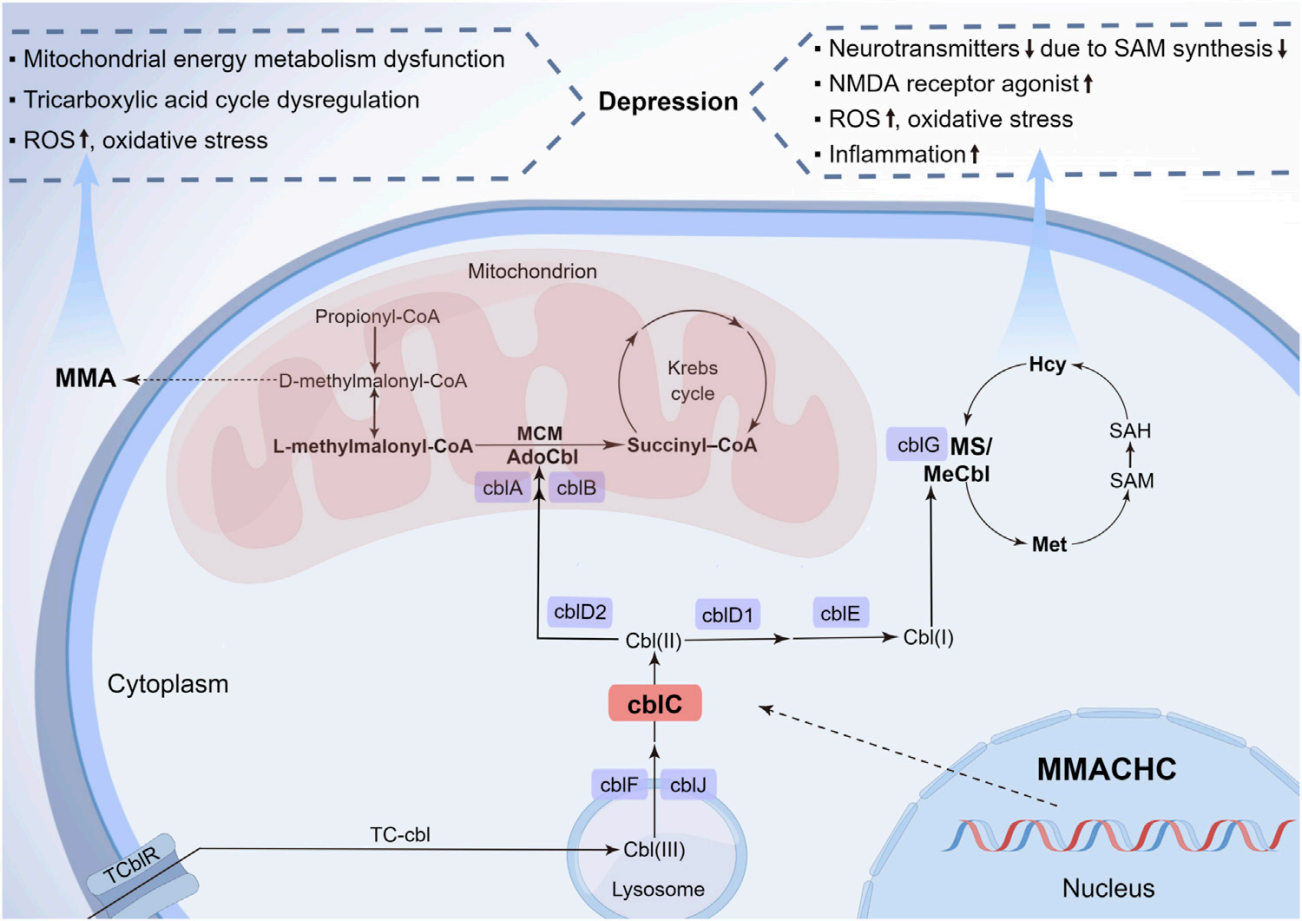
Metabolic pathways of cblC defects and its relationship with depression. Nine complementation group defects of the cobalamin pathways and the metabolic interrelationship of MMA and Hcy are summarized. The neurotoxicity of MMA and Hcy due to the cblC defect is described for their importance in the pathophysiology of depression. cbl, cobalamin; cblA-G, cblJ, cbl complementation group diseases; TC-cbl, transcobalamin–cobalamin complex; TC-cblR, transcobalamin receptor; AdoCbl, 5′-deoxyadenosylcobalamin; MCM, methylmalonyl-CoA mutase; MMA, methylmalonic acid; MeCbl, methylcobalamin; Hcy, homocysteine; Ms, methionine synthesis; Met, methionine; SAM, S-adenosyl-methionine; SAH, S-adenosyl-L-homocysteine; *MMACHC*, gene responsible for methylmalonic acidemia and homocysteinemia; ROS, reactive oxygen species; NMDA, N-methyl-D-aspartate (By Figdraw, ID: UPAOR62009).

Recently, some studies have pointed out that the underlying pathogenesis of depression might involve neurotransmitters and the related amino acid metabolism disorder (Wang et al., 2021), oxidative stress and inflammation (Behl et al., 2022), mitochondrial dysfunction, and energy metabolism disturbance (Weger et al., 2020). Some biochemical alterations in cblC patients may have harmful effects on the development of depression in multiple ways (Figure 2). First, Hcy elevation and vitamin B12 deficiency of cblC defect can result in depression *via* inhibiting the S-adenosyl-methionine-dependent synthesis of variable neurotransmitters and their biological activities, such as catecholamines, namely, dopamine, norepinephrine, epinephrine, and noncatecholamines, namely, serotonin, due to impairment in the methylation pathway (Bhatia and Singh, 2015). Additionally, rising Hcy and cysteine sulfinic acid can produce N-methyl-D-aspartate receptor agonists and reactive oxygen species, both of which have neurotoxic effects on dopaminergic neurons, thus inducing depression (Bhatia and Singh, 2015). Second, the dysfunction of mitochondrial energy metabolism and tricarboxylic acid cycle, caused by the elevated blood MMA level, can reduce neuroplasticity and impair hippocampal neurogenesis (Proctor et al., 2020; Forny et al., 2021), which is widely acknowledged to play a vital role in depression. Third, Hcy, together with MMA, also exacerbates oxidative stress and inflammation that are involved in key depressive pathogenic pathways. Thus, we deemed that the elevated Hcy and MMA levels in cblC patients, especially the former one (Moradi et al., 2021), are linked to depression. This connection is consistent with the fact that depression occurs in late-onset methylmalonic aciduria and homocysteinemia patients more frequently than in early-onset methylmalonic aciduria patients. Understanding the association between depression and cblC disease and its influence on the pathophysiologic mechanism of depression will be helpful in the early diagnosis of cblC disease and the etiology-based treatment of uncommon depression.

Cobalamin, essential for human growth and development, is converted to two active forms: adenosylcobalamin is the coenzyme of mitochondrial methylmalonyl-CoA mutase, converting methylmalonyl-CoA to succinyl-coenzyme A, whose inborn error can cause MMA accumulation due to methylmalonyl-CoA increase; and Mecbl serves as the coenzyme of cytoplasmic methionine synthase, associated with the synthesis of methionine from Hcy, whose congenital disorder can lead to Hcy accumulation and methionine reduction. Details of the metabolic pathways of the cblC defect are shown in Figure 2.

To date, inherited cobalamin deficiencies mainly contain more than 10 types, such as methylmalonyl-CoA deficiency, haptocorrin deficiency, cblA-G, cblJ, cblX, and cblK. Except for cblX, which is X-linked recessive, all the other recognized types are autosomal recessive inheritance. Some types generate a block in the synthesis pathway of both adenosylcobalamin and Mecbl, such as cblC, cblD, cblF, and cblJ, leading to combined methylmalonic aciduria and homocysteinemia (Watkins and Rosenblatt, 2022). Among these types, cblC is the most common inborn disorder. The *MMACHC* mutation was first identified in 2006 (Lerner-Ellis et al., 2006) and consists of nearly 100 variants recorded in the Human Gene Mutation Database (HGMD) up to now, which are responsible for the cblC defect. Many variants differ in RNA stability or residual function of the protein, which may produce, at least, a certain discrepancy in the phenotype and severity of the disease (Maquat, 2004).

Previous published evidence indicated that the mutation spectrum of the *MMACHC* gene seemed to vary in different regions across the world. A study by Wang et al. suggested that c.609G>A (p.W203X), c.658_660delAAG (p.K220del), c.80A>G {p.Q27R [r.(spl?)]}, and c.482 G>A (p.R161Q) mutations were the most common mutations in the Chinese population, while the most frequent mutations in the Caucasian population were c.271dupA (p.R91Kfs∗14), c.394C>T (p.R132X), and c.331C>T (p.R111X) (Wang et al., 2019). However, little difference in the genotype–phenotype correlations of the different regions was observed. Present studies conformably believed that c.271dupA (p.R91Kfs∗14), c.609G>A (p.W203X), and c.331C>T (p.R111X) were mainly related to an early-onset and severe presentation, whilst c.482 G>A (p.R161Q) mainly caused the late onset and mild phenotype in any region (Carrillo-Carrasco et al., 2012; Morel et al., 2006; Wang et al., 2019; Yu et al., 2015).

The mutation in our case was a compound heterozygous mutation in the *MMACHC* gene, that is, c.271dupA (p.R91Kfs∗14) and c.482 G>A (p.R161Q), causing a late onset with mild symptoms, which does not prove the aforementioned correlation. The reason for that may be two points. On one hand, it seems that the compound heterozygotes could result in quite a moderate disease, between the severe early-onset form associated with homozygosity for c.271dupA (p.R91Kfs∗14) and the slight late-onset phenotype related to homozygosity for c.482 G>A (p.R161Q); on the other hand, the late-onset form of the disease usually occurs in patients who possess compound heterozygotes for the c.271dupA (p.R91Kfs∗14) mutation and a missense mutation (Morel et al., 2006). Moreover, the fact that the diverse genetic constitutions in the cblC patients make it difficult to establish the entire connection between genotype and phenotype. Despite this, with the rising awareness of newborn screening and prenatal diagnosis, this genotype–phenotype relationship is still expected to forecast the onset age and disease severity and provide a guide for treatment strategy proposal. Furthermore, case series need to focus on their connection, even on specific clinical manifestations, such as depression, encephalopathy, extremity weakness, gait abnormalities, and seizures, which help to predict the delayed presentation and prognosis.

CblC diagnosis typically depends on either biochemical examination or genetic analysis. Homocysteine in blood or urine, blood amino acids, free carnitine, acylcarnitines, and urine organic acids are vital biochemical assays. Importantly, mass spectrometry and gas chromatography-mass spectrometry are recommended to, respectively, detect blood levels of acylcarnitines, including propionylcarnitine (C3) and acetylcarnitine (C2), and urinary organic acids, including MMA and methylcitric acid. Particularly, using combined mass spectrometry and gas chromatography-mass spectrometry, newborn screening has effective identification ability, boosts early diagnosis, and allows better prognosis (Jin et al., 2021). At the same time, blood vitamin B12, folic acid, and antibody to intrinsic factor need to be detected to exclude the diagnosis of cobalamin disturbance due to secondary etiologies. Finally, genetic sequencing could facilitate the achievement of a definitive diagnosis and detailed genotyping, such as cblC disease based on the *MMACHC* gene mutation. With the improvement of detection means, cblC can be rapidly diagnosed if the physician is conscious of it. However, in the past reported cases, the time to unequivocal diagnosis needs more than 2 months, even 2 years (Wei et al., 2019). Unfortunately, it took us 3 months to make a clear diagnosis, although some points in this immature patient, for example, abnormal homocysteinemia and multiple neuropsychiatric symptoms, have provided a clue of the inherited metabolic disease for us. The major reason for the prolonged time to diagnosis is misdiagnosis or missed diagnosis during the initial visit. Thereby, physicians need to pay more attention to the understanding of cblC symptoms and heterogeneous clinical spectrum and enhance the awareness of disease screening.

Currently, depression is a very common disorder characterized by paroxysm and easy recurrence, and is closely related to living difficulties, abnormal personality development, and suicide in adolescents, which also, inevitably, increase family and social burdens. Although the possibility of a coincidence cannot be completely excluded in this case, the clinical features of this patient’s depression were not typical, for only one attack occurred and the condition improved quickly. Thence, we considered that depression could be a presenting sign of cblC-type methylmalonic aciduria and homocysteinemia, which also indicated that cblC disease might be a cause of depression. Psychiatrists should extend biochemical or genetic analysis looking for this rare disease to those patients with atypical depression, for example, patients with markedly elevated Hcy levels, anemia, intelligence, or fitness decline, which provide crucial clues for genetic metabolic disease. Moreover, extremely elevated serum Hcy levels, anemia, and intelligence or fitness decline provide crucial clues for genetic metabolic disease. In this case, the acylcarnitine profile was normal, which is, indeed, totally beyond our expectations, as the test was conducted after medication treatment for several weeks. It suggested that we conduct the blood and urinary biochemical examinations as early as possible, for an improper test time could lead to completely confusing results. White matter hyperintensity shown in brain MRI is also one of the common image presentations. Fortunately, in this case, when homocysteinemia was detected, the patient was immediately administered a cyanocobalamin and Mecbl supplement, so that she could have a better prognosis. Thence, once cblC is considered, treatment with parenteral cobalamin and betaine is suggested as soon as possible after the biochemical examination, which helps to correct the metabolic disorder. Most notably, hydroxocobalamin is the only form of cobalamin proven to benefit patients with cblC disease, and cyanocobalamin/Mecbl is not recommended as a regular course of treatment (Carrillo-Carrasco et al., 2012; Baumgartner et al., 2014). The treatment of cyanocobalamin/Mecbl can only be applied to the patient with the presence of a partially functional *MMACHC* protein in heterozygosity (Anna et al., 2022) Previous studies and our case study revealed that although the late-onset cblC patient could show improved clinical manifestation and long-life span through active treatments, urine MMA and blood Hcy levels of the patients could not be reduced to normal (Matos et al., 2013; Wei et al., 2019), which indicated that MMA metabolic disturbance would last for a relatively long period, and the delayed complication could follow as well.

These complications include encephalopathy, hydrocephalus (Zhang et al., 2019), optic neuropathy and retinopathy (Alowain et al., 2019), pulmonary hypertension, late-onset diffuse lung disease, cardiorespiratory disease (Liu et al., 2017; Zhang et al., 2020), proteinuria, renal thrombotic microangiopathy, and kidney function decline (Chen et al., 2020; Liu et al., 2017). Thereby, the late-onset cblC patients should be treated perpetually with hydroxocobalamin supplement and routine metabolic monitoring. During the follow-up, the physician needs to pay attention to the signs of these complications and conduct relevant imaging examinations if necessary. Except for the regular therapy, a lot of novel gene therapies, including adenovirus gene addition, genome editing, lentiviral gene therapy, and systemic mRNA therapy, would be effective for permanent long-term correction of metabolic disorders caused by methylmalonyl-CoA mutase gene mutation (Chandler and Venditti, 2019; Schneller et al., 2021). However, it should be noted that there have been no gene therapies available for cblC at present, and more attempts can be conducted to seize the appropriate genomic therapies.

## Conclusion

Collectively, our case report has expanded the cblC clinical symptom spectrum, indicating that the consideration of depression as one kind of the initial sign of cblC would be helpful in the early diagnosis. Literature review about genotype–phenotype correlation and clinical experience could also remarkably improve physicians’ perspectives and patient prognosis for early diagnosis and treatment.

## Data Availability

The datasets for this article are not publicly available due to concerns regarding participant/patient anonymity. Requests to access the datasets should be directed to the corresponding author.
